# Resisting the small cake phenomenon – sharing resources and knowledge makes you rich

**DOI:** 10.3325/cmj.2017.58.93

**Published:** 2017-04

**Authors:** Srećko Gajović

Performing research in an environment lacking the necessary resources, particularly financial resources, is a challenge that researchers sometimes find frustrating and sometimes inspiring. As science needs intellect to thrive, the analytical tools used to solve research questions may as well be used to understand the societal positioning of scientific activities. Researchers may be bewildered by the neglect of science as a source of vital knowledge for the society, but recently, it has been argued that these unfavorable conditions can also be a source of a specific aspect of the scientific excellence (1). The values of national grants can be easily recalculated into the number of publishing fees charged by gold open access journals (2). If an individual research grant per year amounts to less than 10 publishing fees, the grant does not even cover the publication costs of the productive group results, subsequently being of questionable value for both the grant agency and the society.

Even if the underfinancing of research could not be easily explained, scientists could use their intellectual powers to use the resources wisely. One of the major solutions suggested was to join forces, collaborate, and share research equipment and consumables. In other words, even if individual pieces are small, when combined and coordinated, they may become a substantial force and change the game in favor to those who collaborate.

Regrettably, this rarely happens. The fragmentation of resources had already been noticed at the beginning of our careers when, as young PhD students, we pledged to change it when we get our own laboratories. Still, not much has changed. Most of the colleagues claimed their own research identities, preferring to deal with them alone. Others developed intensive international collaborations. Actually, establishing a collaboration with a laboratory abroad turned out to be easier than establishing the collaboration with those around the corner. Thus, the use of equipment belonging to others still remains cumbersome, and even open platforms are not used much. It is so unlike the situation in the research communities with ample resources, where having multiple collaborations, laboratories open for use, and researchers scrolling between departments to use the equipment are a standard.

How may it be possible that those who need collaboration due to their limited resources somehow avoid collaborating, while those with plenty of resources enter collaborations easily and readily share whatever they have available? There are, probably, many possible answers to this question and I would like to share my own. I call it a *small cake phenomenon*.

The small cake phenomenon refers to children’s birthday parties. When a big, beautiful birthday cake is brought in, a dozen or so children quietly line up to get a piece. Those who want another piece can have it without any problems, and the birthday party continues as planned. This is the description of a big cake phenomenon. The small cake phenomenon is just the opposite. There are just as many children, but the birthday cake is very small. The children disappointedly realize that, after the cake is cut it into a dozen or so pieces, each of them would get a ridiculously thin slice. So the fastest child grabs the whole cake, runs to a corner, and starts eating it, facing the wall…

Is there the small cake phenomenon in research environments with low resources? In a sense, there is. Developing a research project with one or two team members, not discussing it with the colleagues, and using only one’s own equipment to run experiments behind the closed doors has some resemblance to the small-cake birthday party described above.

Even if the small cake phenomenon is true, it is not the reason to accept it. The researchers are not children. They have enormous intellectual potential and should indicate the best practices. Actually, those having the courage and strength to join forces will eventually be awarded (3). The scientists deal with the ultimate wealth of humanity – knowledge. Keeping it locked takes away its value. Sharing the knowledge, collaborating, and exchanging ideas is the proper way to create the knowledge with a value, which makes us richer if together than if alone. Actually, whatever the cake size, the point is to enjoy it together.
